# Anakinra-Loaded Sphingomyelin Nanosystems Modulate In Vitro IL-1-Dependent Pro-Tumor Inflammation in Pancreatic Cancer

**DOI:** 10.3390/ijms25158085

**Published:** 2024-07-24

**Authors:** Marcelina Abal-Sanisidro, Michele De Luca, Stefania Roma, Maria Grazia Ceraolo, Maria de la Fuente, Lucia De Monte, Maria Pia Protti

**Affiliations:** 1 Nano-Oncology and Translational Therapeutics Group, Health Research Institute of Santiago de Compostela (IDIS), SERGAS, 15706 Santiago de Compostela, Spain; marce.abal.san@gmail.com; 2 University of Santiago de Compostela (USC), 15782 Santiago de Compostela, Spain; 3 Biomedical Research Networking Center on Oncology (CIBERONC), 28029 Madrid, Spain; 4 Tumor Immunology Unit, Istituto di Ricovero e Cura a Carattere Scientifico (IRCCS) San Raffaele Scientific Institute, 20132 Milan, Italy; michele.deluca@istitutotumori.mi.it (M.D.L.); roma.stefania@hsr.it (S.R.); ceraolo@ingm.org (M.G.C.); demonte.lucia@hsr.it (L.D.M.); 5 Division of Immunology, Transplantation and Infectious Diseases, IRCCS San Raffaele Scientific Institute, 20132 Milan, Italy; 6 DIVERSA Technologies S.L., Edificio Emprendia, Campus Sur, 15782 Santiago de Compostela, Spain

**Keywords:** pancreatic ductal adenocarcinoma, sphingomyelin nanosystems, IL-1, IL-1RA, TSLP, IL-17, IFN-γ

## Abstract

Pancreatic cancer is a very aggressive disease with a dismal prognosis. The tumor microenvironment exerts immunosuppressive activities through the secretion of several cytokines, including interleukin (IL)-1. The IL-1/IL-1 receptor (IL-1R) axis is a key regulator in tumor-promoting T helper (Th)2- and Th17-type inflammation. Th2 cells are differentiated by dendritic cells endowed with Th2-polarizing capability by the thymic stromal lymphopoietin (TSLP) that is secreted by IL-1-activated cancer-associated fibroblasts (CAFs). Th17 cells are differentiated in the presence of IL-1 and other IL-1-regulated cytokines. In pancreatic cancer, the use of a recombinant IL-1R antagonist (IL1RA, anakinra, ANK) in in vitro and in vivo models has shown efficacy in targeting the IL-1/IL-1R pathway. In this study, we have developed sphingomyelin nanosystems (SNs) loaded with ANK (ANK-SNs) to compare their ability to inhibit Th2- and Th17-type inflammation with that of the free drug in vitro. We found that ANK-SNs inhibited TSLP and other pro-tumor cytokines released by CAFs at levels similar to ANK. Importantly, inhibition of IL-17 secretion by Th17 cells, but not of interferon-γ, was significantly higher, and at lower concentrations, with ANK-SNs compared to ANK. Collectively, the use of ANK-SNs might be beneficial in reducing the effective dose of the drug and its toxic effects.

## 1. Introduction

Pancreatic ductal adenocarcinoma (PDAC) is a very aggressive disease with a dismal prognosis (expected to become the second leading cause of cancer mortality by 2030) [1,2]. The tumor microenvironment exerts an immunosuppressive activity, which supports tumor cell growth, invasion, and metastases, and it is sustained by a complex cross-talk between tumor, stromal, and immune cells in the tumor microenvironment [3,4,5,6,7,8,9].

The cytokines of the interleukin (IL)-1 group are inflammatory mediators frequently upregulated in a variety of cancers, and their production is often associated with poor prognosis, with several mechanisms accounting for their pro-tumorigenic effects [10,11].

In PDAC, the activation of *Kras* promotes the production of inflammatory cytokines, including IL-1, which activates NF-κB to promote tumor cell survival and proliferation, increased invasive and metastatic behavior, and angiogenesis [5,12,13,14]. In addition, IL-1 cytokines exert important immunomodulatory functions in the tumor microenvironment, where IL-1α and IL-1β derived from tumor cells and/or tumor-associated macrophages act on IL-1 receptor (IL-1R)-expressing cells, including cancer-associated fibroblasts (CAFs), with the secretion of several tumor-promoting cytokines (including IL-6, IL-8, and transforming growth factor (TGF)-β) [15,16,17,18] and CD4^+^ T helper (Th) cell effectors [19].

In the context of PDAC, we previously reported that the ratio of Th2/Th1 cells is a predictor of poor prognosis after surgery in chemo-naïve patients [20]. Specifically, we found that differentiation of Th2 cells was due to activation of myeloid dendritic cells (DCs) with Th2-polarizing capability that was dependent on the thymic stromal lymphopoietin (TSLP) secreted by tumor-derived IL-1-activated CAFs [20,21,22,23], pointing to a relevant indirect role for IL-1 in driving Th2-type inflammation in PDAC. IL-1 is also a relevant cytokine for human Th17 cell differentiation [24,25,26,27]. Th17 cells were increased in PDAC compared to normal pancreatic tissue [28], and high levels of IL-17 and Th17 cells in the tumor were associated with worse clinical correlates in PDAC patients [28,29,30,31,32,33]. These data highlight the relevance to PDAC of an indirect and a direct role of IL-1 in the differentiation of pro-tumor Th2 and Th17 cells, respectively.

Considering the key role of IL-1 in tumor-promoting inflammation in PDAC, IL-1 inhibition by a recombinant IL-1R antagonist (IL-1RA) (anakinra, ANK) has been tested in in vitro and in vivo preclinical studies, demonstrating that ANK is an effective anti-tumor agent [23,34,35,36]. Based on these results, clinical trials in PDAC patients with metastatic disease as well as in the neoadjuvant setting using ANK in combination with chemotherapy are ongoing/have been planned (NCT02021422, NCT02550327, NCT04926467). Preliminary reports showed the safety and feasibility of this therapeutic approach with toxicities expected from the combination regimen [37,38].

As toxicity due to ANK may represent a problem in these combination regimens, the targeting of ANK to the tumor might impact effective intratumor drug concentrations and reduce toxicities. In this context, the use of nanoparticles for drug delivery may improve drug uptake and/or drug tolerance [39].

Here, we prepared sphingomyelin nanosystems (SNs) loaded with ANK (ANK-SNs) and used them in vitro to test their capacity to inhibit IL-1-induced CAF activation and cytokine secretion and Th17 cell differentiation and function in comparison to the free drug.

## 2. Results

### 2.1. Preparation and Characterization of ANK-SNs

**Figure 1:**
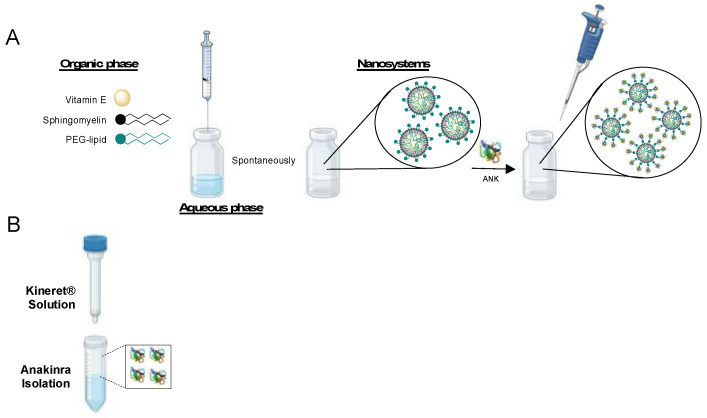
Preparation of the anakinra-loaded sphingomyelin nanosystems (ANK-SNs). (**A**) Schematic representation of the single-step SN preparation by the ethanol injection method, followed by the dropwise addition of ANK solution in PBS at physiological pH. (**B**) ANK isolation from its pharmaceutical form (Kineret^®^) by using desalting columns. (Created with BioRender.com).

We prepared ANK-SNs by adapting the ethanol injection method that we previously described [40,41]. Preparation of unloaded SNs (from now on referred to as SNs), composed of vitamin E (VitE), sphingomyelin (SM), and surfactant lipids, was followed by the dropwise addition of the recombinant protein ANK, as represented in Figure 1A. In order to be loaded in SNs, ANK was isolated from its pharmaceutical form Kineret^®^ by using a desalting column (Figure 1B).

Polyethylene glycol (PEG)ylated lipids have been extensively used to link biomolecules (such as proteins, peptides, and antibodies) to the surfaces of nanostructures [39,42,43]. Indeed, PEG presents features in drug delivery, such as prolonged blood circulation time and reduced potential interactions with blood components (opsonization), that give stealth properties to nanosystems, thus improving their therapeutic efficacy [39,42,43].

To display ANK on the surface of SNs, our strategy was to chemically conjugate it to a PEGylated stearic carbon chain containing a chemically active functional group to perform a covalent reaction (DSPE-PEG(2k)-X; X = NHS, DBCO, or Maleimide) [44,45]. In the case of DSPE-PEG(2k)-DBCO, in order to implement SPAAC click chemistry, ANK was modified with a linker to incorporate the highly reactive group azide (-N_3_) into its structure. For that, we reacted the lysines, which have a terminal group (-NH_2_), with the -NHS ester present in the linker, and N3-ANK modification was verified by high-performance liquid chromatography with mass spectroscopy (HPLC-MS) (Supplementary Figure S1).

**Figure 2:**
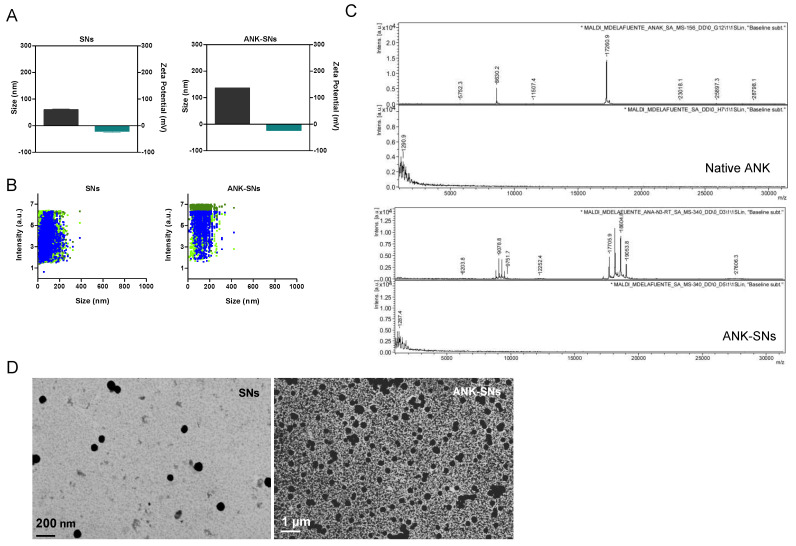
Physicochemical characterization of sphingomyelin nanosystems (SNs) (VitE/SM/NHS) and anakinra-loaded sphingomyelin nanosystems (ANK-SNs) (VitE/SM/NHS/ANK) using several analytical techniques. (**A**) Size (nm) (black columns) and surface charge (mV) (turquoise columns) of SNs and ANK-SNs, measured by dynamic light scattering (DLS) and dynamic light scattering (LDA). Data are expressed as mean ± SD (at least n = 5). (**B**) Complementary physicochemical characterization by nanoparticle tracking analysis (NTA) of SNs and ANK-SNs (n ± 5) is represented as size vs. light scattering intensity (arbitrary unit a.u.). (**C**) Matrix-assisted laser desorption/ionization (MALDI) time-of-flight (TOF) analysis of native ANK (calculated exact mass of 17.260 kDa) and ANK-SNs (calculated mean mass ~20 kDa). (**D**) Representative field emission scanning electron microscopy (FESEM) images of SNs (left) and ANK-SNs (right). SN scale bar: 200 nm. ANK-SN scale bar: 1 μm.

We obtained three SN formulations, differing one from the other in the surfactant lipid linker used in its preparation (i.e., VitE/SM/DBCO, VitE/SM/Maleimide, and VitE/SM/NHS), and we performed their physicochemical characterization in terms of size and surface charge by dynamic light scattering (DLS) and laser Doppler anemometry (LDA). All formulations showed a small particle size, a monodisperse population, and a negative zeta potential (Table 1 and Figure 2A). Interestingly, particles of this size (<200 nm) are more likely to accumulate in tumors thanks to the enhanced permeation retention effect [46]. Furthermore, nanosystems with negatively charged surfaces can reduce undesirable clearance by the reticuloendothelial system and improve blood compatibility, making their delivery to tumor sites more efficient [47]. The negatively charged surface also makes them more biocompatible in comparison to positively charged nanosystems, which interact with cell structures by electrostatic interactions [47,48]. The percentage of ANK loading was highest (92%) for the formulation composed of VitE/SM/DBCO/ANK, whereas it was similar, i.e., 72% and 75%, for the formulations VitE/SM/Maleimide/ANK and VitE/SM/NHS/ANK, respectively (Table 1).

As in the in vitro assays used to address the nanosystem biological functions (see below), after preliminary testing with all three formulations, VitE/SM/NHS/ANK was chosen for subsequent experiments; we performed full characterization of the nanosystems with this formulation. In addition to DLS, nanosystems were characterized by nanoparticle tracking analysis (NTA). NTA is based on the combination of laser light scattering microscopy with a charge-coupled device camera that visualizes and analyzes individual tracks of nanoparticles moving under Brownian motion, allowing the calculation of particle size and concentration [49,50]. Compared to SNs, ANK-SNs presented a higher mean particle size as well as span value due to the loading of the ANK protein and the interaction of the nanoparticles with the phosphate-buffered saline (PBS) (Figure 2B and Table 2). D-values D10, D50, and D90 representative of the particle size diameter at 10, 50, and 90% cumulative distribution are reported in Table 2. Collectively, these results well correlate with DLS data.

We also performed matrix-assisted laser desorption/ionization (MALDI) time-of-flight (TOF) spectrometry to confirm an increase in ANK mass due to the covalent bond to DSPE-PEG(2k)-NHS (estimated ~2.8 kDa) (Supplementary Figure S2). Indeed, for ANK-SNs, we observed an increment in mass (~20 KDa) compared to the one expected for the native form of ANK (17.3 kDa) (Figure 2C). Other nanosystem components, such as VitE and SM, were not detected by this technique, as they are not coupled to the protein.

Lastly, we used field emission scanning electron microscopy (FESEM) to check the particle distribution and the spatial disposition. As shown in Figure 2D, representative images clearly indicated the characteristic morphology and spherical shape of the SNs, as we previously reported [40]. In the case of ANK-SNs, we observed a slight increment in the particle size (Figure 2D, right panel), already reported by DLS and NTA data. The spherical distribution did not seem to be affected by the loading of the ANK protein, pointing out the good stability of the nanosystems.

Collectively, as verified and confirmed by DLS, NTA, MALDI-TOF, and FESEM analyses, ANK protein was successful and efficiently loaded at the SN surface with an expected increment in size of about 60–65 nm.

#### 2.1.1. ANK-SN Stability over Time and in Biorelevant Media

**Figure 3:**
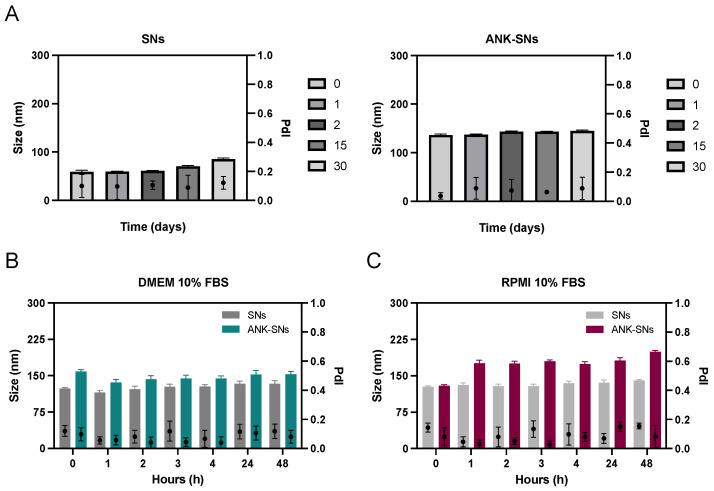
Stability in size and polydispersity index (PdI) of sphingomyelin nanosystems (SNs) and anakinra-loaded sphingomyelin nanosystems (ANK-SNs) over time. (**A**) Stability up to 30 days of SNs and ANK-SNs stored at 4 °C. Stability upon incubation up to 48 h in DMEM (**B**), and RPMI (**C**), supplemented with 10% FBS at 37 °C and under orbital stirring (300 rpm).

We assessed the colloidal stability of nanosystems under different storage conditions using DLS. We showed that size and polydispersity index (PdI) for both SNs and ANK-SNs were stable for at least one month (Figure 3A). Stability was also tested in different culture conditions. SNs and ANK-SNs were incubated in Dulbecco’s modified eagle medium (DMEM) and RPMI culture media supplemented with 10% fetal bovine serum (FBS) at 37 °C, and particle size and PdI were monitored for 48 hours (h). We found that the two parameters remained stable in culture with both media (Figure 3B,C). This stability can be explained by the adsorption of serum proteins to the nanosystem surface due to charge balance. ANK-SNs also seemed to be stabilized thanks to the electrostatic interaction between ANK and serum proteins.

Collectively, we found that the nanosystems maintained their colloidal properties over time and in different culture media, and we proceeded to study their internalization and cytotoxicity in cell systems in vitro.

#### 2.1.2. In Vitro Internalization and Viability Assays of the Nanosystems in Tumor Cells

Before using ANK-SNs in cell systems relevant to the study of immunomodulation of Th2 and Th17 pro-tumor inflammation in PDAC, we challenged our nanosystems with PDAC cells and looked at their internalization and cytotoxic potential. 

**Figure 4:**
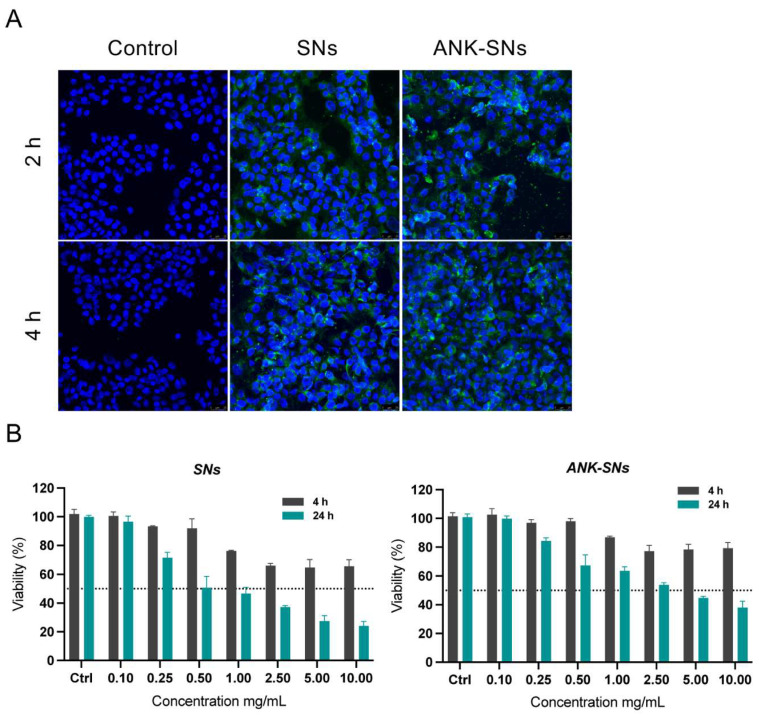
Internalization and cytotoxicity assays of nanosystems using pancreatic ductal adenocarcinoma (PDAC) cells. (**A**) Representative confocal microscopy images after 2 h and 4 h treatment in L3.6p cells. Fluorescent-labeled TopFluor^®^ sphingomyelin nanosystems (SNs) and TopFluor^®^ anakinra-loaded sphingomyelin nanosystems (ANK-SNs) are in green. Cell nuclei stained with Hoechst 33342 are in blue. (**B**) Cell viability determined by Alamar Blue^TM^ assay after treatment with SNs and ANK-SNs (0.1 mg/mL to 10 mg/mL) for 4 h and 24 h. Data are expressed as means ± SD. The dotted line is set at 50% of viability.

We performed internalization studies by confocal microscopy using the PDAC cell line L3.6pl. For these experiments, nanosystems were prepared as described above but using fluorescent-labeled TopFluor^®^ SNs. As shown in Figure 4A, we detected high fluorescent signals at the two time points tested (i.e., 2 h and 4 h). Internalization seemed more efficient when we used ANK-SNs compared to SNs, possibly related to the interaction of the ANK protein with components present at the cell membrane. Interestingly, we previously found *IL1R1* mRNA expression in several PDAC cell lines and high *IL1R1* expression was found in the L3.6pl precursor cell line L3.3 (The Human Protein Atlas), suggesting specific interaction of ANK with its receptor, namely IL-1R1.

For cell viability assays, we treated the L3.6pl cells for 4 h and 24 h at increasing concentrations of nanosystems (0.1 mg/mL to 10 mg/mL corresponding to 2.2 μg/mL to 220 μg/mL of ANK), and cytotoxicity was revealed by Alamar Blue^TM^ fluorescent assay. As shown in Figure 4B, at lower concentrations (0.10–0.50 mg/mL), the nanosystems showed little cytotoxicity. At higher concentrations, decreased viability was seen in both nanosystems, although cytotoxicity was lower for ANK-SNs compared to SNs. These results defined the toxic concentrations (i.e., 0.25–0.5 mg/mL) used to guide the following experiments.

### 2.2. ANK and ANK-SNs Are Equivalent in Down-Modulating Cytokine Secretion by IL-1-Activated CAFs

We then moved to compare the effect of ANK versus ANK-SNs in inhibiting the secretion of tumor-promoting cytokines in the tumor microenvironment that depends on IL-1/IL-1R signaling. CAFs are a relevant cell population in the PDAC stroma that express IL-1R1 [51], and when stimulated with IL-1α and/or IL-1β, they secrete several pro-tumor cytokines [36,52,53], including TSLP, which is responsible for pro-tumor Th2 inflammation in PDAC [20,23]. Indeed, we previously reported that CAFs obtained from primary PDAC samples and activated in vitro with IL-1α and IL-1β (or IL-1-containing supernatants from PDAC microenvironment cultures) release TSLP, which in turn activates myeloid DCs with Th2-polarizing capability [20]. Importantly, we also found that interfering in vitro and in vivo with the IL-1/IL-1R signaling pathway with ANK inhibited TSLP secretion by activated CAFs [23].

**Figure 5:**
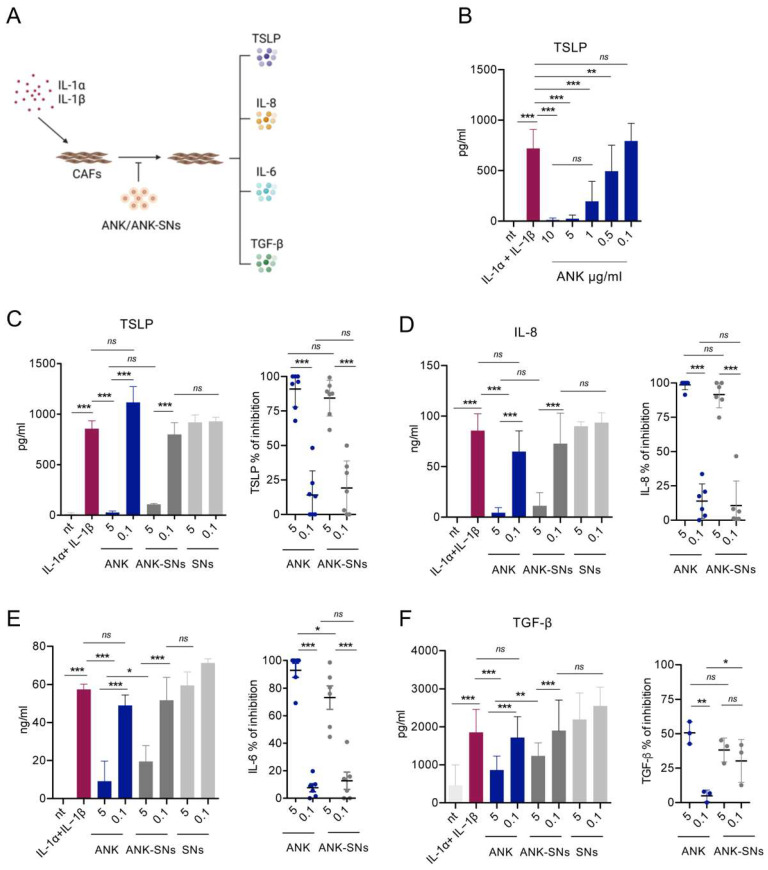
Anakinra (ANK) and anakinra-loaded sphingomyelin nanosystems (ANK-SNs) are equivalent in down-modulating cytokine secretion by IL-1-activated cancer-associated fibroblasts (CAFs). (**A**) In vitro experimental model. CAFs were treated with recombinant IL-1α + IL-1β, mimicking tumor-derived IL-1, in the absence or in the presence of ANK, ANK-SNs, or SNs. Secretion of TSLP, IL-8, IL-6, and TGF-β was measured after culture. (Created with BioRender.com). (**B**) Dose–response curve to detect the best concentrations of ANK for inhibiting TSLP secretion by CAFs (n = 3). ANK was added at the indicated concentrations. Untreated CAFs were used as negative (nt) control; IL-1α + IL-1β-treated CAFs were used as positive (IL-1α + IL-1β) control. (**C**–**F**) ANK, ANK-SNs, and SNs (VitE/SM/NHS formulation), tested for their capacity to down-modulate cytokine secretion, were added at the indicated concentrations and based on the titration curve shown in (**B**). Negative and positive controls used were as in B. (**C**) TSLP. *Left*, TSLP secretion with controls (n = 2). *Right*, TSLP percentage inhibition of cumulative experiments (n = 7). (**D**) IL-8. *Left*, IL-8 secretion with controls (n = 2). *Right*, IL-8 percentage inhibition of cumulative experiments (n = 6). (**E**) IL-6. *Left*, IL-6 secretion with controls (n = 2)*. Right*, IL-6 percentage inhibition of cumulative experiments (n = 6). (**F**) TGF-β. *Left*, TGF-β secretion with controls (n = 2). *Right*, TGF-β percentage inhibition of cumulative experiments (n = 3). Data are mean ± SEM from the indicated number (n) of independent experiments. Significance was calculated by one-way ANOVA test and Newman–Keuls post-test. Values were considered significantly different for * *p* < 0.05, ** *p* < 0.01, *** *p* < 0.001; ns = not significant.

In this study, we tested the inhibitory activity of ANK-SNs compared to free ANK by using the in vitro system depicted in Figure 5A. CAFs were stimulated with IL-1α + IL-1β in the absence and in the presence of ANK, ANK-SNs, or SNs (i.e., to evaluate the basal level of cytokine modulation by the SNs alone), and after 2 days, the supernatant was collected for the detection of the tumor-promoting TSLP, IL-8, IL-6, and TGF-β cytokines [20,23,36,52,53]. To identify the optimal concentrations of ANK to use in these experiments, we created a dose–response curve with different ANK concentrations and used the release of TSLP as a readout. We found that 5 μg/mL gave the maximum inhibitory effect, whereas 0.1 μg/mL did not show inhibitory effects (Figure 5B), and we decided to use these two concentrations in the following experiments. We then tested the three nanosystem formulations (i.e., VitE/SM/DBCO, VitE/SM/Maleimide, and VitE/SM/NHS) to detect the best-performing one(s) using the two selected concentrations. We found that at 5 μg/mL, the VitE/SM/NHS/ANK and VitE/SM/DBCO/ANK formulations inhibited TSLP release at levels comparable to ANK, whereas VitE/SM/Maleimide was much less efficient (Figure 5C, left panel, and Supplementary Figure S3A). In addition, when we considered the effect of the SNs alone and in comparison to the other formulations, VitE/SM/NHS did not show any stimulatory effect (Figure 5C, left panel, and Supplementary Figure S3A), and it was chosen for the following experiments. In the same experimental setting, we also measured the release of pro-tumor IL-8, IL-6, and TGF-β. In the case of IL-8, the results were very similar to TSLP (Figure 5D, left panel), whereas in the case of IL-6 (Figure 5E, left panel) and TGF-β (Figure 5F, left panel), the inhibition induced by ANK-SNs at 5 μg/mL was slightly inferior to the one obtained with ANK. The results of a larger set of experiments verified that ANK and ANK-SNs equally inhibited the release of the different cytokines (Figure 5C–F, right panels).

We confirmed in CAFs that treatment with the nanosystems did not induce relevant cytotoxicity at the concentrations (i.e., 0.1 and 5 μg/mL) used in experiments shown in Figure 5B (Supplementary Figure S4A). In addition, an analysis of internalization of the nanosystems by confocal microscopy showed that down-modulation of the IL-1R1 expression at the CAF surface after treatment was significantly higher with ANK-SNs compared to SNs (Supplementary Figure S5A), demonstrating the specific interaction of IL-1R1 with ANK-SNs at the CAF surface.

Collectively, we found that ANK-SNs did not have cytotoxic effects, were efficiently internalized through specific interaction at the cell surface with IL-1R1, and down-modulated the secretion by CAFs of TSLP, IL-8, IL-6, and TGF-β, thus dampening their tumor-promoting functions.

### 2.3. ANK-SNs Are Superior to Free ANK in Down-Modulating the Secretion of IL-17, but Not of Interferon-γ (IFN-γ), by In Vitro Differentiated Th17 Cells

**Figure 6:**
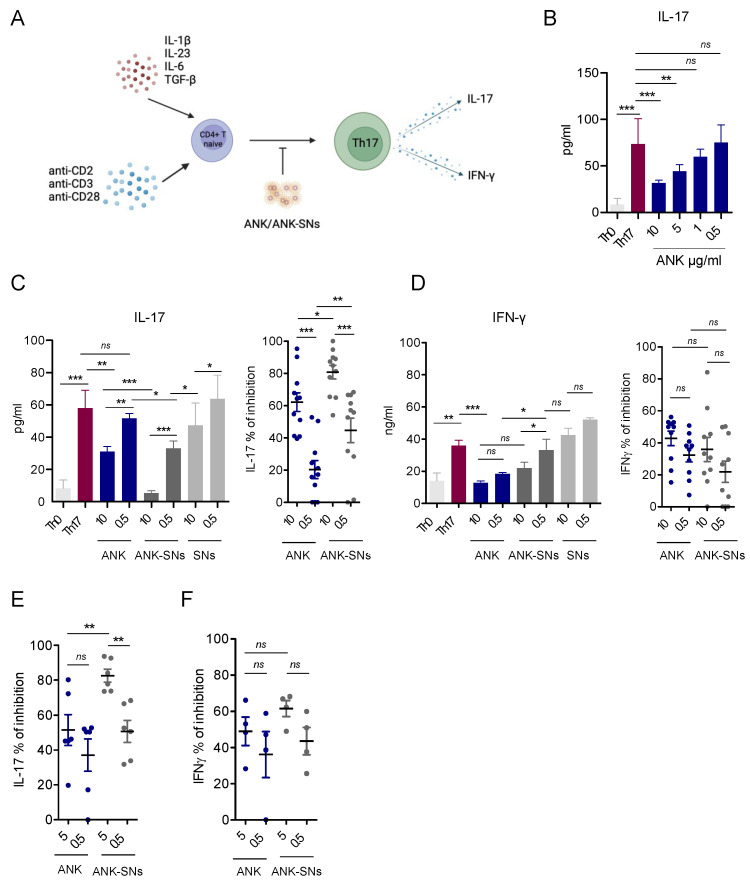
Anakinra-loaded sphingomyelin nanosystems (ANK-SNs) are superior to free anakinra (ANK) in down-modulating the secretion of IL-17, but not of IFN-γ, by in vitro differentiated Th17 cells. (**A**) In vitro experimental model. Naïve CD4^+^ T cells were differentiated towards Th17 cells by treatment with anti-CD2/CD3/CD28-coated beads and IL-1β, IL-23, IL-6, and TGF-β, and in the absence or in the presence of ANK, ANK-SNs, or SNs. On day 5, Th17 cells were collected and restimulated with anti-CD2/CD3/CD28-coated beads, and IL-17 and IFN-γ secretion levels were measured by ELISA. (Created with BioRender.com). (**B**) Dose–response curve to determine the best concentrations of ANK for inhibiting IL-17 secretion in Th17 cells (n = 3). ANK was added at the indicated concentrations. Th0 (i.e., naïve CD4^+^ T cells activated with anti-CD2/CD3/CD28-coated beads only) were used as a negative control. Th17 cells were used as a positive control. (**C**,**D**) ANK, ANK-SNs, and SNs (VitE/SM/NHS formulation), tested for their capacity to down-modulate cytokine secretion by Th17 cells, were added at the indicated concentrations and based on the titration curve shown in B. (**C**) IL-17. *Left*, IL-17 secretion with controls (n = 2). *Right*, IL-17 percentage inhibition of cumulative experiments (n = 11) (**D**) IFN-γ. *Left*, IFN-γ secretion with controls (n = 2). *Right*, IFN-γ percentage inhibition of cumulative experiments (n = 10). (**E**) IL-17 percentage inhibition of cumulative experiments using 5 μg/mL of ANK/ANK-SNs (n = 6). (**F**) IFN-γ percentage inhibition of cumulative experiments using 5 μg/mL of ANK/ANK-SNs (n = 4). Data are mean ± SEM from the indicated number (n) of independent experiments. Significance was calculated by one-way ANOVA test and Newman–Keuls post-test. Values were considered significantly different for * *p* < 0.05, ** *p* < 0.01, *** *p* < 0.001.

Th17-type inflammation has been reported to favor tumor progression in PDAC [28,29,31,32,33]. Due to the role of IL-1 in the differentiation of human Th17 cells [24,25,26,27], we evaluated the effect of ANK-SNs added in culture during in vitro Th17 differentiation from naïve CD4^+^ T cells. We followed the experimental scheme described in [27] and depicted in Figure 6A. Naïve CD4^+^ T cells were activated with anti-CD2, anti-CD3, and anti-CD28 antibody-coated beads and cultured in the presence of the polarizing cytokines (i.e., IL-1β, IL-23, IL-6, and TGF-β) and in the absence or the presence of ANK, ANK-SNs, and SNs. After 5 days of culture, Th17 cells were restimulated with the antibody-coated beads for 24 h, and the supernatant was collected for IL-17 and IFN-γ detection. To identify the best concentration of ANK to be used to inhibit IL-17 secretion by Th17 cells, we created dose–response titration curves (Figure 6B). Based on these experiments, we decided to use 10 μg/mL (with maximum inhibitory effect) and 0.5 μg/mL (without inhibitory effect) for the initial testing with the three nanosystem formulations. Similar to the results obtained with CAFs, the VitE/SM/NHS/ANK formulation showed the best performance (Figure 6C and Supplementary Figure S3B). Indeed, VitE/SM/DBCO/ANK was inferior to VitE/SM/NHS/ANK in inhibiting IL-17 secretion, and in the case of VitE/SM/Maleimide/ANK, an inhibitory function was already present with SNs alone (Supplementary Figure S3B, right). Importantly, VitE/SM/NHS/ANK at both 10 and 0.5 μg/mL induced inhibition of IL-17 secretion that was significantly higher compared with the one obtained with ANK (Figure 6C, left and right). As Th17 cells, in addition to IL-17, may secrete IFN-γ, which is a relevant anti-tumor cytokine [54], we measured the release of this cytokine in the same experimental setting. Both ANK and ANK-SNs inhibited IFN-γ secretion at comparable levels, although inhibition was much lower compared to that of IL-17 (Figure 6D, left and right). Lastly, as we found that 5 μg/mL was the best ANK-SN concentration to use in the CAF experiments, we verified the efficacy of this dose also in the Th17 model. We also confirmed that at 5 μg/mL, ANK-SNs were superior to ANK in inhibiting IL-17 secretion by Th17 cells (Figure 6E), and importantly at levels even higher than the ones obtained with ANK at 10 μg/mL (median level 80% compared to 60%) (Figure 6E and Figure 6C, right panel). As reported above, no significant difference between ANK-SNs and ANK in inhibiting IFN-γ secretion by Th17 was observed (Figure 6F).

We confirmed in Th17 cells that nanosystems did not induce relevant cytotoxicity in any conditions and concentrations used, when tested with both colorimetric and flow cytometry assays (Supplementary Figure S4B–D). Confocal microscopy showed internalization of the nanosystems with down-modulation of the IL-1R1 expression on Th17 cells that was significantly higher after treatment with ANK-SNs compared to SNs (Supplementary Figure S5B), demonstrating a specific interaction of ANK-SNs with IL-1R1.

Collectively, we found that nanosystems did not affect Th17 cell viability, were internalized through ANK/IL-1R1 interaction, and exerted their modulatory effects primarily by inhibiting pro-tumor IL-17, while preserving anti-tumor IFN-γ.

## 3. Discussion

Protein-based therapeutics are an important class of medicines, accounting for more than 130 pharmaceutics approved for clinical use in the European Union and the United States, to treat a wide variety of human conditions, including cancer [55,56,57]. However, they suffer from enzymatic degradation, short half-life, and physicochemical instability with a loss of bioactivity, hampering their clinical efficacy [39]. To overcome these issues, their delivery can be improved by combining therapeutic proteins with nanotechnology, and lipidic and polymeric nanocarriers have been described for this purpose [39,58,59].

In this work, we used lipidic nanosystems that enable covalent binding of the protein ANK to the modified hydrophobic carbon chains disposed onto the surface. Previous extensive characterization of these nanosystems, composed of the two natural compounds, i.e., VitE and SM, revealed suitable physicochemical properties, very high biocompatibility in vitro and in vivo, and colloidal stability during storage and in biological media, all relevant properties for clinical translation [40]. All these properties were confirmed for the nanosystems loaded with ANK, described here.

ANK has been used to treat autoimmune and autoinflammatory disorders with clinical benefit, especially for autoinflammatory diseases [60]. However, because of its short half-life, ANK needs daily administration to maintain its efficacy, with relevant local side effects at the subcutaneous injection site that may lead to drug discontinuation [61]. The ANK-SN formulations described here might help in targeting the drug to the relevant site of action (i.e., the tumor) with increased efficacy and clinical benefit, but also increased tolerability.

Due to the role of IL-1 in tumor progression [10,11], ANK has been tested in preclinical in vitro and in vivo settings with encouraging results [23,34,35,36], and clinical trials have planned the incorporation of ANK into treatment protocols [62]. ANK was used in small pilot studies of combination regimens in advanced metastatic colorectal cancer with long-lasting tumor stabilization [63] and in HER2-negative metastatic breast cancer with a reduction in or stabilization of the tumor volume [64]. In PDAC, ANK was tested in combination with the highly toxic FOLFIRINOX regimen in advanced disease [37], and in combination with gemcitabine, nab-paclitaxel, and cisplatin in patients with non-metastatic disease prior to resection, resulting in reduced local cancer spread compared with historical controls receiving chemotherapy alone [38]. Collectively, these clinical trials revealed potential clinical benefit for ANK, and loading of the drug in the nanosystems described here might greatly increase the therapeutic efficacy while reducing side effects, not only in PDAC but also in other tumors where the IL-1/IL-1R axis is relevant.

The aim of the present study was to evaluate the efficacy of ANK as a free drug compared to the drug loaded in the nanosystems in two in vitro models of Th2- and Th17-type inflammation relevant to PDAC progression.

Concerning Th2 inflammation, we showed that ANK-SNs, at both 5 and 0.1 μg/mL, were equal to the free drug in inhibiting TSLP secretion by CAFs, suggesting that the drug loaded in the nanosystems maintained its efficacy. In addition, other tumor-promoting cytokines released by CAFs were also inhibited by ANK-SNs, including IL-8, IL-6, and TGF-β (although at levels slightly inferior to ANK). Interestingly, these two last cytokines are also relevant inducers of Th17 differentiation [27], suggesting a comprehensive effect in the tumor microenvironment.

Concerning Th17 cell differentiation, we found that ANK-SNs were superior to the free drug in inhibiting IL-17 secretion at both 10 and 5 μg/mL. In addition, the inhibition of IL-17 secretion by Th17 cells with ANK-SNs at 5 μg/mL reached a mean of about 80% that was still superior to the inhibition obtained with ANK alone at 10 μg/mL, meaning that a relevant effect was observed at a much lower drug concentration. This is important when considering drug toxicity.

Another important aspect is the performance of ANK-SNs when considering the IFN-γ secreted by Th17 cells. In different cancer contexts, Th17 cells have been described with either pro- or anti-tumor functions [65]. This paradoxical behavior of Th17 cells has been attributed to their plasticity potential that enables their conversion into other types of CD4^+^ T cells or their acquisition of polyfunctional activity, such as Th17/T regulatory and Th17/Th1. In these last polyfunctional Th17 cells, the release of IFN-γ might contribute to anti-tumor activity. In our in vitro system, we found that, although the inhibition of IL-1 signaling by both ANK and ANK-SNs reduced the secretion of both IL-17 and IFN-γ, the inhibition of IFN-γ was not significantly different between the two drug compositions (mean 40%) and was similar at all concentrations tested. This means that ANK-SNs compared to ANK were more efficacious in inhibiting IL-17 secretion while maintaining an equal much lower level of inhibition of anti-tumor IFN-γ.

In conclusion, using two in vitro model systems of primary CAFs and differentiated Th17 cells, we have demonstrated that ANK loaded in nanosystems maintains (and importantly, ANK-SNs are more efficacious than the free drug even at lower concentrations) their capacity to downregulate the secretion of cytokines that are indirectly or directly relevant in PDAC for the establishment of Th2- and Th17-type inflammation, respectively.

We showed that these nanosystems do not present significant toxicity in vitro and are stable in culture, thus guaranteeing their biocompatibility and encouraging safe application in medical practice. Altogether, our results encourage further in vivo testing of ANK loaded in these nanosystems, which could offer advantages for patients with PDAC and other neoplastic diseases where treatment with ANK has already shown positive clinical effects.

Mouse models recapitulating the tumor-promoting Th2- and Th17-type inflammation, as reported in the human disease, have been described [29,66,67], and they could offer good models for exploring the new pharmaceutical preparations, comprising any additional/different side effects compared to the ones described for ANK, described here. 

As IL-1 family cytokines have been described in mouse models to play important roles in regulating host–microbiome dialogue at barrier sites [68], in future studies, understanding whether and how therapeutic inhibition of these cytokines may impact microbiome dysbiosis might provide further opportunities to improve patients’ outcome.

## 4. Materials and Methods

### 4.1. Materials

Vitamin E (VitE, DL-α-Tocopherol) was purchased from Sigma (Merck, Darmstadt, Germany, EU). Sphingomyelin (SM, Lipoid E SM) was kindly provided by Lipoid GmbH (Ludwigshafen, Germany, EU). Surfactant lipids, being DSPE-PEG(2k)-X (X = DBCO, Maleimide, NHS), were obtained from Avanti^®^ Polar Lipids (Alabaster, AL, USA). Fluorescent labeling lipid, C11 TopFluor^®^ Sphingomyelin, was also acquired from Avanti^®^ Polar Lipids. Azido-dPEG^®^_8_-NHS ester was provided by Merck (Darmstadt, Germany, EU). HPLC-grade ethanol (EtOH) and dimethyl sulfoxide were purchased from Thermo Fisher Scientific (Rockford, IL, USA). DMEM was obtained from Sigma, RPMI 1640 Medium, FBS and penicillin/streptomycin from Thermo Fisher Scientific, Iscove’s modified Dulbecco’s medium (IMDM) and X-VIVO^TM^ 20 Medium both from Lonza (Walkersville, MD, USA).

### 4.2. Preparation of ANK and Azide-Modified ANK (N3-ANK) and Characterization

The recombinant protein ANK was purified from the other components present in the pharmaceutical form Kineret^®^, namely salts, using a desalting column (CentriPure 10, emp Biotech GmbH, Berlin, Germany, EU), and eluted with PBS (2 mM, pH 7.4), following the manufacturer instructions. The concentration of the obtained ANK protein was determined by the BCA Protein Assay Kit (Thermo Fisher Scientific, Rockford, IL, USA).

The ANK protein (0.058 µmol) was also modified with Azido-dPEG^®^_8_-NHS linker (1.16 µmol) (Sigma, Merck, Darmstadt, Germany, EU) in PBS (10 mM). The reaction was performed at room temperature (RT) for 6 h, with storage at 4 °C. Linker excess was removed by using spin filters (Amicon^®^ Ultra Centrifugal Filters, 10 k, 0.5 mL, Merck, Darmstadt, Germany, EU) in a refrigerated microcentrifuge (5417R; Eppendorf), as previous reported [40]. N3-ANK modification was characterized by HPLC-MS (TimsTof Pro, Bruker, Billerica, MA, USA; coupled to the Elute UHPLC) by dissolving 5 pmol of sample in ultrapure water. The analysis method used was positive electrospray ionization, acquisition range 500–3000 *m*/*z*, chromatographic separation with column C4, and the mobile phases employed were A, water/formic acid 0.1%, and B, acetonitrile/formic acid 0.1%; the chromatographic separation time was 15 minutes (min). MALDI-TOF analysis was carried out in Ultraflex III TOF/TOF (Bruker, Billerica, MA, USA); the analysis method chosen was MALDI linear mode ionization using a sinapinic acid matrix.

### 4.3. Preparation of SNs, ANK-SNs, and TopFluor^®^-SM-Labeled ANK-SNs

SNs and ANK-SNs composed of VitE, SM, and surfactant lipids were prepared by adapting the ethanol injection method, as previously described [40]. Briefly, 5 mg of VitE, 0.5 mg of SM, and 0.05 mg of surfactant lipids were dissolved in 100 μL of ethanol. SNs were instantaneously formed by injecting the organic phase into 0.9 mL of ultrapure water, at RT. ANK-SNs were then formed by dropwise adding ANK solution (14 µM) in PBS under magnetic stirring for up to 5 min and incubated at RT for 6 h followed by an overnight incubation at 4 °C. 

Fluorescent-labeled SNs were prepared by admixing TopFluor^®^-SM (4.5 μg) with the other compounds of the organic phase (final volume of 100 μL). The organic phase was then injected in 0.9 mL of ultrapure water and immediately formed at RT.

### 4.4. Physicochemical Characterization of the Nanosystems

Particle size and PdI were determined by DLS and Z-potential values by LDA, using a Zetasizer NanoZS^®^ (Malvern Instruments, Worcestershire, UK). Samples were diluted to 1:10 with ultrapure water, and the measurements were performed at 25 °C with a detection angle of 173°. SN and ANK-SN formulations were evaluated in biological media to ensure the maintenance of their properties during in vitro assays. Formulations were incubated at 37 °C with 10% FBS-supplemented DMEM and RPMI 1640 under orbital shaking. Additionally, extended characterization was performed by NTA. For NTA, the nanosystems were diluted at 1:1000 (*v*/*v*) in ultrapure water using a NanoSight NS3000 system (Malvern Instrument, UK) with a laser operating at λ = 488 nm and 200 mW power. Data collection was settled with 3 repeats/60 s capture time, with both shutter and gain manually determined for each sample. NTA 2.0 Build 127 software was used for measurement and subsequent data analysis.

SNs and ANK-SNs were analyzed by MALDI-TOF. The analysis was conducted using Ultraflex III TOF/TOF (Bruker) equipment; the method used was MALDI linear mode ionization using a sinapinic acid matrix with previous sample ZipTip desalting.

SNs and ANK-SNs were evaluated for particle size and morphology distribution by FESEM, using a ZEISS FESEM ULTRA Plus microscope (Carl Zeiss Micro Imaging, GmbH, Germany, EU) configured with InLens and STEM mode, operating at 20 kV. First, 20 µL (0.55 mg/mL) of a filtered nanosystem was stained with phosphotungstic acid (2% *w*/*v*) at a ratio of 1:1. Next, 10 µL was placed on a carbon-coated grid and left for 30 s, twice. The grid was allowed to dry and then repeatedly washed with filtered ultrapure water. Finally, it was let dry until its visualization.

### 4.5. Establishment of CAF Cell Lines

Primary cultures of CAFs were obtained from PDAC surgical samples, as we previously described [20]. Briefly, tumor pieces were put in culture in IMDM medium (Lonza, Walkersville, MD, USA) plus 10% FBS, and CAFs obtained by outgrowth. CAFs were characterized for expression of the fibroblast-activation protein (FAP) marker and the absence of expression of the EpCAM epithelial marker by flow cytometry, as described below. 

CAFs at 3rd-4th culture passage were stably transfected to express the human telomerase reverse transcriptase gene (hTERT) by lentiviral infection using hTERT Cell Immortalization KIT (ALSTEM, Richmond, CA, USA), according to manufacturer’s instructions. Seventy-two hours after infection, puromycin (Sigma) selection was applied at 1.5 μg/mL final concentration. Puromycin-resistant colonies were checked for hTERT expression by real-time qPCR. CAF cell lines were periodically tested for Mycoplasma contamination using the MycoBlue Mycoplasma Detector kit (D101, Vazyme, Nanjing, China).

### 4.6. Effects of Nanosystems on Cytokine Secretion by CAFs

CAFs were seeded at 3 × 10^4^ cells/well in IMDM 10% FBS in flat-bottom 96-well plates and starved overnight without serum. The next day, the medium was replaced with IMDM 2% FBS alone or with 20 ng/mL of each recombinant human IL-1α + IL-1β (R&D Systems, Minneapolis, MN, USA). In the inhibition experiments, ANK (Kineret^®^, Amgen Europe, Breda, The Netherlands), ANK-SNs and SNs were added at the indicated concentrations. After 4 h of incubation, the medium was replaced with IMDM 2% FBS for 72 h. Cell viability was tested as described below and is reported in Supplementary Figure S4A. TSLP, IL-6, IL-8, and TGF-β secretion in CAF supernatants was measured by ELISA (TSLP and TGF-β, R&D Systems; IL-6 and IL-8, Mabtech, Nacka Strand, Sweden, EU).

### 4.7. Effects of Nanosystems on Th17 Cell Differentiation and Cytokine Secretion

Peripheral blood mononuclear cells (PBMCs) were obtained by Ficoll-Hypaque (Cytiva, Malborough, MA, USA) gradient stratification from healthy donor buffy coats. Naïve CD4^+^ T cells were magnetically isolated from PBMCs after two rounds of Naïve CD4^+^ T cell Isolation Kit (Miltenyi Biotec, Bergish Gladbach, Germany, EU) and plated at 1 × 10^6^ cells/well in 48-well plates containing X-VIVO 20 in the presence of anti-CD2-, anti-CD3-, and anti-CD28-coated beads (Miltenyi Biotec, Bergish Gladbach, Germany, EU). The following cytokines were added: IL-1β (10 ng/mL) (R&D Systems), IL-6 (20 ng/mL) (PeproTech, Cranbury, NJ, USA), TGF-β (1 ng/mL) (PeproTech), and IL-23 (50 ng/mL) (PeproTech or R&D Systems). Th0 cells were obtained by stimulation of purified naïve CD4^+^ T cells with the antibody-coated beads in the absence of the polarizing cytokines. In inhibition experiments, ANK, ANK-SNs, and SNs were added at the indicated concentrations. After 5 days, cells were collected and washed extensively, and their viability was determined as described below and is reported in Supplementary Figure S4B–D. CD4^+^ T cells (1 × 10^6^/mL) were restimulated for 24 h with anti-CD2-, anti-CD3-, and anti-CD28-coated beads (one bead per cell). The secretion of IL-17 and IFN-γ in culture supernatants was detected by ELISA (Mabtech, Nacka Strand, Sweden, EU).

### 4.8. Cell Viability

The L3.6pl PDAC cell line, originally described in [69], was kindly supplied by Dr. Bruno Sainz Jr (Madrid, Spain, EU). L3.6pl cells were cultured in RPMI 1640 media (Invitrogen, Thermo Fisher Scientific, Waltham, MA, USA) containing 10% FBS and 50 units/mL penicillin/streptomycin in a humidified incubator at 37 °C, and cultures were tested for Mycoplasma contamination periodically, as above. L3.pl6 cells were seeded at a density of 2.5 × 10^4^ cell/well into black-wall clear-bottom 96-well plates (Sigma) and incubated in the absence or the presence of increasing concentration of SNs or ANK-SNs for 4 h and 24 h. Cell viability was determined using a 1:10 dilution AlamarBlue^TM^ assay. Fluorescence was read using a Victor Nivo^TM^ multimode plate reader (Thermo Fisher Scientific, Waltham, MA, USA) at 530 nm excitation and 590 nm emission. Data were normalized to negative control 100% viability, and the reagent itself was used as a fluorescence background.

CAFs were stimulated with pro-inflammatory cytokines, and Th17 cells were differentiated and cultured both in the absence and in the presence of inhibitory stimuli, as described above. At the end of stimulation/differentiation, CAFs were washed with PBS 100 μL of IMDM 10% FBS culture medium was replaced, and Th17 cells were collected and cultured in 100 μL X-VIVO 20. Cell viability was evaluated by the addition of 20 μL/well of CellTiter 96 Aqueous One Solution Cell Proliferation Assay (PROMEGA, Madison, WI, USA) for 4 h. The absorbance was recorded at 490 nm and 650 nm to subtract the background. The viability of Th17 cells was also tested by flow cytometry; see below.

### 4.9. Flow Cytometry

Th17 cells were harvested after in vitro restimulation for 24 h with antibody-coated beads, as described above, and stained with anti-human CD4 (BD Pharmingen^TM^ FITC mouse anti-human CD4, cat. 555346) and diluted 1:1000 with Invitrogen™ LIVE/DEAD™ Fixable Aqua Dead Cell Stain Kit (Invitrogen, Thermo Fisher Scientific, Waltham, MA, USA). Cell viability was assessed by flow cytometry; data were acquired with BD FACSCanto. A gating strategy was adapted to avoid autofluorescence derived from antibody-coated beads (Supplementary Figure S4C). CAFs were characterized by staining with anti-FAP antibody (APC-conjugated mouse anti-human FAP antibody, R&D Systems, cat. FAB3715A) and anti-EpCAM antibody (PE-conjugated mouse anti-human EpCAM/CD326 antibody, BD Biosciences, San Jose, CA, USA, cat. 347198), following the manufacturer’s instructions, and data were acquired as above.

### 4.10. Confocal Microscopy

Internalization of nanosystems in L3.6pl cells was evaluated by confocal laser microscopy (Leica Microscope SP8^®^, Wetzlar, Germany, EU). First, 6 × 10^4^ cells were seeded in an 8-well chamber slide plate and grew overnight at 37 °C. Fluorescent-labeled TopFluor^®^-SM SNs and fluorescent-labeled TopFluor^®^-SM ANK-SNs (0.2 mg/mL) were added for 2 h. The treatment was then removed, cells were fixed for 15 min with 4% paraformaldehyde (PFA) (Thermo Fisher Scientific), and cell nuclei were stained with Hoechst 33342 (Thermo Fisher Scientific).

For CAF experiments, 1 × 10^5^ cells/well were plated on a glass coverslip, previously treated with 0.01% Poly-L-lysine solution (Sigma), and starved overnight. The next day, CAFs were stimulated with IL-1α + IL-1β, as described above, and treated with ± ANK or fluorescent-labeled TopFluor^®^-SM SNs or fluorescent-labeled TopFluor^®^-SM ANK-SNs for 4 h, fixed with 4% PFA (Thermo Fisher Scientific) in PBS for 10 min at RT and washed with PBS. Treated CAFs were permeabilized with 0,1% saponin in dH_2_O for 15 min. The coverslips were then blocked overnight at 4 °C with PBS 2% BSA (Sigma). Successively, a first incubation with anti-IL-1R1 primary antibody (Invitrogen, Thermo Fisher Scientific, Waltham, MA, USA) for 1 h at RT and a secondary incubation with Alexa Fluor 546 (Invitrogen, Thermo Fisher Scientific, Waltham, MA, USA) for 2 h at RT were applied. Hoechst 33342 (Thermo Fisher Scientific, Waltham, MA, USA) was added to mark the nuclei, and the coverslips were mounted in glycerol. Cells were imaged with an Olympus FV3000 confocal laser scanning microscope.

For Th17 cell experiments, CD4^+^ naïve T cells were magnetically isolated from PBMCs and plated at 1 × 10^6^ cells/well on a glass coverslip, previously treated with 0,01% Poly-L-lysine solution (Sigma), and stimulated, as described above, with IL-1β, IL-6, TGF-β, and IL-23 in the presence of anti-CD2-, anti-CD3-, and anti-CD28-coated beads ± fluorescent-labeled TopFluor^®^-SM SNs or fluorescent-labeled TopFluor^®^-SM ANK-SNs for 5 days. Th17 cells were then fixed with 4% PFA (Thermo Fisher Scientific) in PBS for 10 min at RT, washed with PBS, and blocked overnight at 4 °C with PBS 2% BSA. Successively, a first incubation with anti-IL-1R1 primary antibody (Invitrogen, Thermo Fisher Scientific, Waltham, MA, USA) for 1 h at RT and a secondary incubation with Alexa Fluor 546 (Invitrogen) for 2 h at RT were applied. Hoechst 33342 (Thermo Fisher Scientific, Waltham, MA, USA was added to mark the nuclei, and the coverslips were mounted in glycerol. Cells are imaged with an Olympus FV3000 confocal laser scanning microscope.

For IL-1R1 quantification, area coverage on the cellular surface was determined using ImageJ Software 1.53k. Z-plans were initially acquired and successively stacked to obtain a single image that was converted to 8-bit. After the threshold was applied, the area of the image covered by the fluorochrome linked to IL-1R1 was measured.

### 4.11. Statistics

Differences between groups were statistically determined using a one-way ANOVA test and Neuman–Keuls post-test. The percentage of inhibition was calculated by considering the values of the positive controls in the case of ANK and SNs in the case of ANK-SNs as 100% expression. All statistical analyses were performed using GraphPad Prism (Version 6.0 software) (GraphPadSoftware, San Diego, CA, USA). A *p* value < 0.05 was considered to be significant.

## Figures and Tables

**Table 1:** Physicochemical characterization of sphingomyelin nanosystems (SNs) (composed of VitE/SM/PEG-lipid-X) and anakinra-loaded sphingomyelin nanosystems (ANK-SNs) (composed of VitE/SM/PEG-lipid-X-ANK) measured by dynamic light scattering (DLS) and laser Doppler anemometry (LDA) *.

Formulation	Size (nm)	PdI	ZP (mV)	% ANK
**VitE/SM/DBCO**	55 ± 1	<0.2	−26 ± 1	-
**VitE/SM/DBCO/ANK**	162 ± 2	<0.1	−28 ± 1	92 ± 2
**VitE/SM/Maleimide** **VitE/SM/Maleimide/ANK**	61 ± 2 163 ± 7	<0.2 0.2	−28 ± 1 −22 ± 1	- 72 ± 1
**VitE/SM/NHS** **VitE/SM/NHS/ANK**	60 ± 3 136 ± 2	0.2 0.2	−21 ± 4 −23 ± 1	- 75 ± 2

* Results are expressed as mean ± SD, n = 5. Abbreviations used: nm = nanometer, PdI = polydispersity index, ZP = zeta potential in millivolts (mV). Percentage of ANK associated with SNs was quantified by BCA protein assay.

**Table 2:** Physicochemical properties of sphingomyelin nanosystems (SNs) (composed of VitE/SM/PEG-lipid-NHS) and anakinra-loaded sphingomyelin nanosystems (ANK-SNs) (composed of VitE/SM/PEG-lipid-NHS-ANK) determined by nanoparticle tracking analysis (NTA) *.

Formulation	Mean Size (nm)	D10	D50	D90	Span	Concentration (Particles/mL)
**SNs**	87 ± 2	49 ± 5	85 ± 1	129 ± 3	0.7	5.4 × 10^11^
**ANK-SNs**	133 ± 6	96 ± 2	132 ± 2	178 ± 3	0.9	6.2 × 10^11^

* Mean particle size (diameter), D-values (D10, D50, D90), calculated SPAN value and sample concentration in particles per milliliter (particle/mL) (mean ± SD, n = 5).

## Data Availability

The data presented in thus study are available upon request to the corresponding authors.

## Supplementary Materials

The following supporting information can be downloaded at https://www.mdpi.com/article/10.3390/ijms25158085/s1.

## Abbreviations

ANK: anakinra; ANK-SNs, anakinra loaded-SNs; BSA, bovine serum albumin; CAFs, cancer-associated fibroblasts; DCs, dendritic cells; DLS, dynamic light scattering; DMEM, Dulbecco’s modified eagle medium; FAP, fibroblast-activation protein; FBS, fetal bovine serum; FESEM, field emission scanning microscopy; h, hour/hours, HPLC-MS, high-performance liquid chromatography with mass spectroscopy; hTERT, human telomerase reverse transcriptase gene; IFN-γ; interferon-γ; IL, interleukin; IL-1R, IL-1 receptor; IL-1RA, IL-1R antagonist; IMDM; Iscove’s modified Dulbecco’s medium; LDA, laser Doppler anemometry; MALDI-TOF, matrix-assisted laser desorption/ionization time-of-flight; min, minutes; NTA, nanoparticle tracking analysis; PBMCs, peripheral blood mononuclear cells; PBS, phosphate-buffered saline; PDAC, pancreatic ductal adenocarcinoma; PdI, polydispersity index; PEG, polyethylene glycol; PFA, paraformaldehyde; RT, room temperature; SM, sphingomyelin; SNs, unloaded-nanosystems; TGF-β, transforming growth factor-β; Th, T helper; TSLP, thymic stromal lymphopoietin; VitE, vitamin E.

## References

[1] Hidalgo M. (2010). Pancreatic cancer. N. Engl. J. Med..

[2] Rahib L., Smith B.D., Aizenberg R., Rosenzweig A.B., Fleshman J.M., Matrisian L.M. (2014). Projecting cancer incidence and deaths to 2030: The unexpected burden of thyroid, liver, and pancreas cancers in the United States. Cancer Res..

[3] Feig C., Gopinathan A., Neesse A., Chan D.S., Cook N., Tuveson D.A. (2012). The pancreas cancer microenvironment. Clin. Cancer Res..

[4] Vonderheide R.H., Bayne L.J. (2013). Inflammatory networks and immune surveillance of pancreatic carcinoma. Curr. Opin. Immunol..

[5] Wormann S.M., Diakopoulos K.N., Lesina M., Algul H. (2014). The immune network in pancreatic cancer development and progression. Oncogene.

[6] Protti M.P., De Monte L. (2013). Immune infiltrates as predictive markers of survival in pancreatic cancer patients. Front. Physiol..

[7] Zheng L., Xue J., Jaffee E.M., Habtezion A. (2013). Role of immune cells and immune-based therapies in pancreatitis and pancreatic ductal adenocarcinoma. Gastroenterology.

[8] Leinwand J., Miller G. (2020). Regulation and modulation of antitumor immunity in pancreatic cancer. Nat. Immunol..

[9] Huber M., Brehm C.U., Gress T.M., Buchholz M., Alashkar Alhamwe B., von Strandmann E.P., Slater E.P., Bartsch J.W., Bauer C., Lauth M. (2020). The Immune Microenvironment in Pancreatic Cancer. Int. J. Mol. Sci..

[10] Garlanda C., Mantovani A. (2021). Interleukin-1 in tumor progression, therapy, and prevention. Cancer Cell.

[11] Dosch A.R., Singh S., Nagathihalli N.S., Datta J., Merchant N.B. (2022). Interleukin-1 signaling in solid organ malignancies. Biochim. Biophys. Acta Rev. Cancer.

[12] Gukovsky I., Li N., Todoric J., Gukovskaya A., Karin M. (2013). Inflammation, autophagy, and obesity: Common features in the pathogenesis of pancreatitis and pancreatic cancer. Gastroenterology.

[13] Hausmann S., Kong B., Michalski C., Erkan M., Friess H. (2014). The role of inflammation in pancreatic cancer. Adv. Exp. Med.Biol..

[14] Zambirinis C.P., Pushalkar S., Saxena D., Miller G. (2014). Pancreatic cancer, inflammation, and microbiome. Cancer J..

[15] Zhang T., Ren Y., Yang P., Wang J., Zhou H. (2022). Cancer-associated fibroblasts in pancreatic ductal adenocarcinoma. Cell Death Dis..

[16] Sun Q., Zhang B., Hu Q., Qin Y., Xu W., Liu W., Yu X., Xu J. (2018). The impact of cancer-associated fibroblasts on major hallmarks of pancreatic cancer. Theranostics.

[17] Huang H., Brekken R.A. (2020). Recent advances in understanding cancer-associated fibroblasts in pancreatic cancer. Am. J. Physiol. Cell Physiol..

[18] Geng X., Chen H., Zhao L., Hu J., Yang W., Li G., Cheng C., Zhao Z., Zhang T., Li L. (2021). Cancer-Associated Fibroblast (CAF) Heterogeneity and Targeting Therapy of CAFs in Pancreatic Cancer. Front. Cell Dev. Biol..

[19] Jain A., Song R., Wakeland E.K., Pasare C. (2018). T cell-intrinsic IL-1R signaling licenses effector cytokine production by memory CD4 T cells. Nat. Commun..

[20] De Monte L., Reni M., Tassi E., Clavenna D., Papa I., Recalde H., Braga M., Di Carlo V., Doglioni C., Protti M.P. (2011). Intratumor T helper type 2 cell infiltrate correlates with cancer-associated fibroblast thymic stromal lymphopoietin production and reduced survival in pancreatic cancer. J. Exp. Med..

[21] Protti M.P., De Monte L. (2012). Cross-talk within the tumor microenvironment mediates Th2-type inflammation in pancreatic cancer. Oncoimmunology.

[22] De Monte L., Woermann S., Brunetto E., Heltai S., Magliacane G., Reni M., Paganoni A.M., Recalde H., Mondino A., Falconi M. (2016). Basophil recruitment into tumor draining lymph nodes correlates with Th2 inflammation and reduced survival in pancreatic cancer patients. Cancer Res..

[23] Brunetto E., De Monte L., Balzano G., Camisa B., Laino V., Riba M., Heltai S., Bianchi M., Bordignon C., Falconi M. (2019). The IL-1/IL-1 receptor axis and tumor cell released inflammasome adaptor ASC are key regulators of TSLP secretion by cancer associated fibroblasts in pancreatic cancer. J. Immunother. Cancer.

[24] Chung Y., Chang S.H., Martinez G.J., Yang X.O., Nurieva R., Kang H.S., Ma L., Watowich S.S., Jetten A.M., Tian Q. (2009). Critical regulation of early Th17 cell differentiation by interleukin-1 signaling. Immunity.

[25] Revu S., Wu J., Henkel M., Rittenhouse N., Menk A., Delgoffe G.M., Poholek A.C., McGeachy M.J. (2018). IL-23 and IL-1beta Drive Human Th17 Cell Differentiation and Metabolic Reprogramming in Absence of CD28 Costimulation. Cell Rep..

[26] Lee W.W., Kang S.W., Choi J., Lee S.H., Shah K., Eynon E.E., Flavell R.A., Kang I. (2010). Regulating human Th17 cells via differential expression of IL-1 receptor. Blood.

[27] Volpe E., Servant N., Zollinger R., Bogiatzi S.I., Hupe P., Barillot E., Soumelis V. (2008). A critical function for transforming growth factor-beta, interleukin 23 and proinflammatory cytokines in driving and modulating human T(H)-17 responses. Nat. Immunol..

[28] He S., Fei M., Wu Y., Zheng D., Wan D., Wang L., Li D. (2011). Distribution and clinical significance of Th17 cells in the tumor microenvironment and peripheral blood of pancreatic cancer patients. Int. J. Mol. Sci..

[29] McAllister F., Bailey J.M., Alsina J., Nirschl C.J., Sharma R., Fan H., Rattigan Y., Roeser J.C., Lankapalli R.H., Zhang H. (2014). Oncogenic Kras activates a hematopoietic-to-epithelial IL-17 signaling axis in preinvasive pancreatic neoplasia. Cancer Cell.

[30] Chellappa S., Hugenschmidt H., Hagness M., Line P.D., Labori K.J., Wiedswang G., Tasken K., Aandahl E.M. (2016). Regulatory T cells that co-express RORgammat and FOXP3 are pro-inflammatory and immunosuppressive and expand in human pancreatic cancer. Oncoimmunology.

[31] Lang C., Wang J., Chen L. (2017). CD25-expressing Th17 cells mediate CD8(+) T cell suppression in CTLA-4 dependent mechanisms in pancreatic ductal adenocarcinoma. Exp. Cell Res..

[32] Barilla R.M., Diskin B., Caso R.C., Lee K.B., Mohan N., Buttar C., Adam S., Sekendiz Z., Wang J., Salas R.D. (2019). Specialized dendritic cells induce tumor-promoting IL-10(+)IL-17(+) FoxP3(neg) regulatory CD4(+) T cells in pancreatic carcinoma. Nat. Commun..

[33] Khan I.A., Singh N., Gunjan D., Gopi S., Dash N., Gupta S., Saraya A. (2023). Increased circulating Th17 cell populations in patients with pancreatic ductal adenocarcinoma. Immunogenetics.

[34] Zhuang Z., Ju H.Q., Aguilar M., Gocho T., Li H., Iida T., Lee H., Fan X., Zhou H., Ling J. (2016). IL1 Receptor Antagonist Inhibits Pancreatic Cancer Growth by Abrogating NF-kappaB Activation. Clin. Cancer Res..

[35] Takahashi R., Macchini M., Sunagawa M., Jiang Z., Tanaka T., Valenti G., Renz B.W., White R.A., Hayakawa Y., Westphalen C.B. (2021). Interleukin-1beta-induced pancreatitis promotes pancreatic ductal adenocarcinoma via B lymphocyte-mediated immune suppression. Gut.

[36] Dosch A.R., Singh M., Dai X., Mehra S., Silva I.C., Bianchi A., Srinivasan S., Gao Z., Ban Y., Chen X. (2021). Targeting Tumor-Stromal IL6/STAT3 Signaling through IL1 Receptor Inhibition in Pancreatic Cancer. Mol. Cancer Ther..

[37] Whiteley A., Becerra C., McCollum D., Paulson A.S., Goel A. (2016). A pilot, non-randomized evaluation of the safety of anakinra plus FOLFIRINOX in metastatic pancreatic ductal adenocarcinoma patients. J. Clin. Oncol..

[38] Becerra C., Paulson A.S., Cavaness K.M., Celinski S. (2018). Gemcitabine, nab-paclitaxel, cisplatin, and anakinra (AGAP) treatment in patients with non-metastatic pancreatic ductal adenocarcinoma (PDAC). J. Clin.Oncol..

[39] Viegas C.S., Seck F., Fonte P. (2022). An insight on lipid nanoparticles for therapeutic proteins delivery. J. Drug. Deliver. Technol..

[40] Bouzo B.L., Calvelo M., Martin-Pastor M., Garcia-Fandino R., de la Fuente M. (2020). In Vitro-In Silico Modeling Approach to Rationally Designed Simple and Versatile Drug Delivery Systems. J. Phys. Chem. B.

[41] Bouzo B.L., Lores S., Jatal R., Alijas S., Alonso M.J., Conejos-Sanchez I., de la Fuente M. (2021). Sphingomyelin nanosystems loaded with uroguanylin and etoposide for treating metastatic colorectal cancer. Sci. Rep..

[42] Harris J.M., Chess R.B. (2003). Effect of pegylation on pharmaceuticals. Nat. Rev. Drug Discov..

[43] Suk J.S., Xu Q., Kim N., Hanes J., Ensign L.M. (2016). PEGylation as a strategy for improving nanoparticle-based drug and gene delivery. Adv. Drug Deliv. Rev..

[44] Wang L., Jiang R., Wang L., Liu Y., Sun X.L. (2016). Preparation of chain-end clickable recombinant protein and its bio-orthogonal modification. Bioorg. Chem..

[45] Yoon H.Y., Lee D., Lim D.K., Koo H., Kim K. (2022). Copper-Free Click Chemistry: Applications in Drug Delivery, Cell Tracking, and Tissue Engineering. Adv. Mater..

[46] Fang J., Nakamura H., Maeda H. (2011). The EPR effect: Unique features of tumor blood vessels for drug delivery, factors involved, and limitations and augmentation of the effect. Adv. Drug Deliv. Rev..

[47] Duan X., Li Y. (2013). Physicochemical characteristics of nanoparticles affect circulation, biodistribution, cellular internalization, and trafficking. Small.

[48] Cheng Q., Wei T., Farbiak L., Johnson L.T., Dilliard S.A., Siegwart D.J. (2020). Selective organ targeting (SORT) nanoparticles for tissue-specific mRNA delivery and CRISPR-Cas gene editing. Nat. Nanotechnol..

[49] Filipe V., Hawe A., Jiskoot W. (2010). Critical evaluation of Nanoparticle Tracking Analysis (NTA) by NanoSight for the measurement of nanoparticles and protein aggregates. Pharm. Res..

[50] Kim A., Ng W.B., Bernt W., Cho N.J. (2019). Validation of Size Estimation of Nanoparticle Tracking Analysis on Polydisperse Macromolecule Assembly. Sci. Rep..

[51] Elyada E., Bolisetty M., Laise P., Flynn W.F., Courtois E.T., Burkhart R.A., Teinor J.A., Belleau P., Biffi G., Lucito M.S. (2019). Cross-Species Single-Cell Analysis of Pancreatic Ductal Adenocarcinoma Reveals Antigen-Presenting Cancer-Associated Fibroblasts. Cancer Discov..

[52] Tjomsland V., Spangeus A., Valila J., Sandstrom P., Borch K., Druid H., Falkmer S., Falkmer U., Messmer D., Larsson M. (2011). Interleukin 1alpha sustains the expression of inflammatory factors in human pancreatic cancer microenvironment by targeting cancer-associated fibroblasts. Neoplasia.

[53] Kennel K., Bozlar M., De Valk A.F., Greten F.R. (2023). Cancer-Associated Fibroblasts in Inflammation and Antitumor Immunity. Clin. Cancer Res..

[54] Burke J.D., Young H.A. (2019). IFN-gamma: A cytokine at the right time, is in the right place. Semin. Immunol..

[55] Leader B., Baca Q.J., Golan D.E. (2008). Protein therapeutics: A summary and pharmacological classification. Nat. Rev. Drug Discov..

[56] Dimitrov D.S. (2012). Therapeutic proteins. Methods Mol. Biol..

[57] Lagasse H.A., Alexaki A., Simhadri V.L., Katagiri N.H., Jankowski W., Sauna Z.E., Kimchi-Sarfaty C. (2017). Recent advances in (therapeutic protein) drug development. F1000Research.

[58] Sivadasan D., Sultan M.H., Madkhali O., Almoshari Y., Thangavel N. (2021). Polymeric Lipid Hybrid Nanoparticles (PLNs) as Emerging Drug Delivery Platform-A Comprehensive Review of Their Properties, Preparation Methods, and Therapeutic Applications. Pharmaceutics.

[59] Dilliard S.A., Siegwart D.J. (2023). Passive, active and endogenous organ-targeted lipid and polymer nanoparticles for delivery of genetic drugs. Nat. Rev. Mater..

[60] Cavalli G., Colafrancesco S., Emmi G., Imazio M., Lopalco G., Maggio M.C., Sota J., Dinarello C.A. (2021). Interleukin 1alpha: A comprehensive review on the role of IL-1alpha in the pathogenesis and treatment of autoimmune and inflammatory diseases. Autoimmun. Rev..

[61] Bettiol A., Lopalco G., Emmi G., Cantarini L., Urban M.L., Vitale A., Denora N., Lopalco A., Cutrignelli A., Lopedota A. (2019). Unveiling the Efficacy, Safety, and Tolerability of Anti-Interleukin-1 Treatment in Monogenic and Multifactorial Autoinflammatory Diseases. Int. J. Mol. Sci..

[62] Fang Z., Jiang J., Zheng X. (2023). Interleukin-1 receptor antagonist: An alternative therapy for cancer treatment. Life Sci..

[63] Isambert N., Hervieu A., Rebe C., Hennequin A., Borg C., Zanetta S., Chevriaux A., Richard C., Derangere V., Limagne E. (2018). Fluorouracil and bevacizumab plus anakinra for patients with metastatic colorectal cancer refractory to standard therapies (IRAFU): A single-arm phase 2 study. Oncoimmunology.

[64] Wu T.C., Xu K., Martinek J., Young R.R., Banchereau R., George J., Turner J., Kim K.I., Zurawski S., Wang X. (2018). IL1 Receptor Antagonist Controls Transcriptional Signature of Inflammation in Patients with Metastatic Breast Cancer. Cancer Res..

[65] Marques H.S., de Brito B.B., da Silva F.A.F., Santos M.L.C., de Souza J.C.B., Correia T.M.L., Lopes L.W., Neres N.S.M., Dorea R., Dantas A.C.S. (2021). Relationship between Th17 immune response and cancer. World J. Clin. Oncol..

[66] Ochi A., Nguyen A.H., Bedrosian A.S., Mushlin H.M., Zarbakhsh S., Barilla R., Zambirinis C.P., Fallon N.C., Rehman A., Pylayeva-Gupta Y. (2012). MyD88 inhibition amplifies dendritic cell capacity to promote pancreatic carcinogenesis via Th2 cells. J. Exp. Med..

[67] Dey P., Li J., Zhang J., Chaurasiya S., Strom A., Wang H., Liao W.T., Cavallaro F., Denz P., Bernard V. (2020). Oncogenic KRAS-Driven Metabolic Reprogramming in Pancreatic Cancer Cells Utilizes Cytokines from the Tumor Microenvironment. Cancer Discov..

[68] Narros-Fernandez P., Chomanahalli Basavarajappa S., Walsh P.T. (2024). Interleukin-1 family cytokines at the crossroads of microbiome regulation in barrier health and disease. FEBS J..

[69] Bruns C.J., Harbison M.T., Kuniyasu H., Eue I., Fidler I.J. (1999). In vivo selection and characterization of metastatic variants from human pancreatic adenocarcinoma by using orthotopic implantation in nude mice. Neoplasia.

