# Supplementary material for: Anakinra-Loaded Sphingomyelin Nanosystems Modulate In Vitro IL-1-Dependent Pro-Tumor Inflammation in Pancreatic Cancer
Source: Int J Mol Sci. 2024 Jul 24;25(15):8085. doi: 10.3390/ijms25158085 (PMC11312284; doi:10.3390/ijms25158085)
Supplement: Supplementary file 1 [file ijms-25-08085-s001.zip › ijms-3043552-supplementary.pdf]

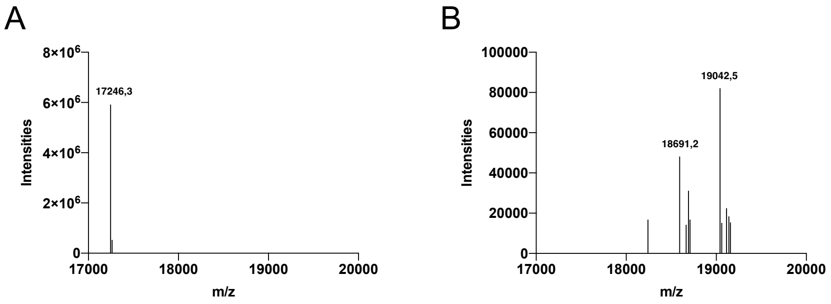

Supplementary Figure S1. Characterization of Azide-modified ANK (N3-ANK) with Azido-dPEG(R)8-NHS by HPLC-MS spectrometry. (**A**) native ANK protein. (**B**) N3-ANK modified protein.

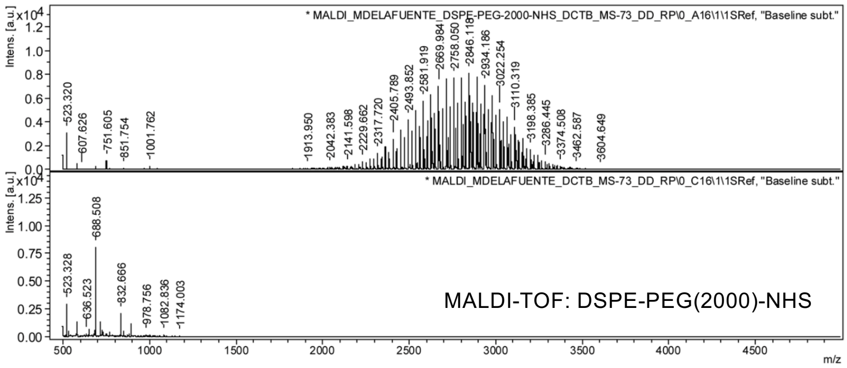

Supplementary Figure S2. MALDI-TOF analysis of DSPE-PEG(2k)-NHS to calculate its mass (2.8 kDa) corresponding to the one given by the manufacturer.

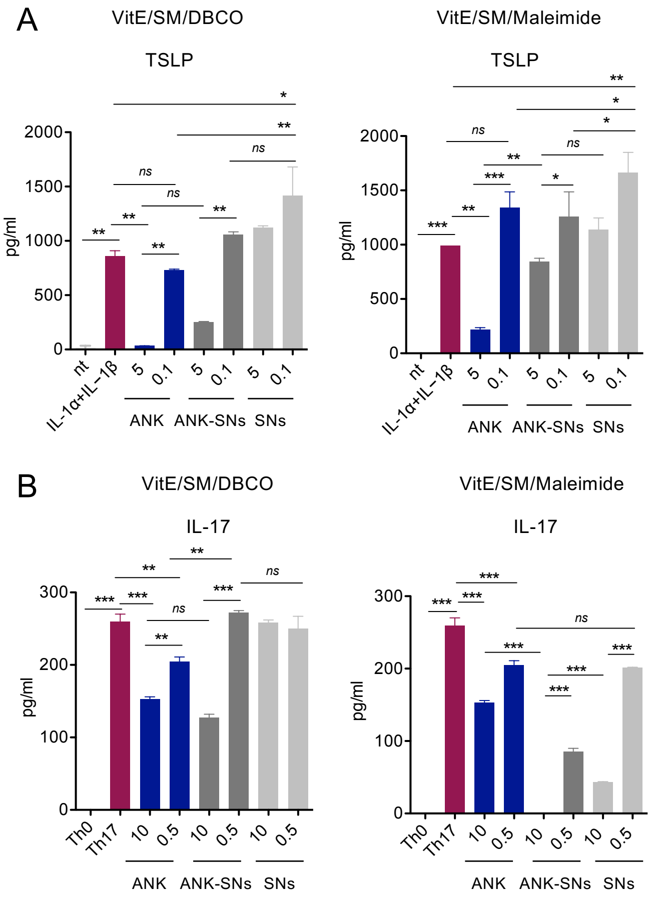

Supplementary Figure S3. Modulation of cytokine secretion by CAFs and Th17 cells in the presence of ANK, ANK-SNs, and SNs using two different nanosystem formulations (VitE/SM/DBCO and VitE/SM/Maleimide). (**A**) TSLP secretion by IL-1-treated CAFs. (**B**) IL-17 secretion by Th17 cells

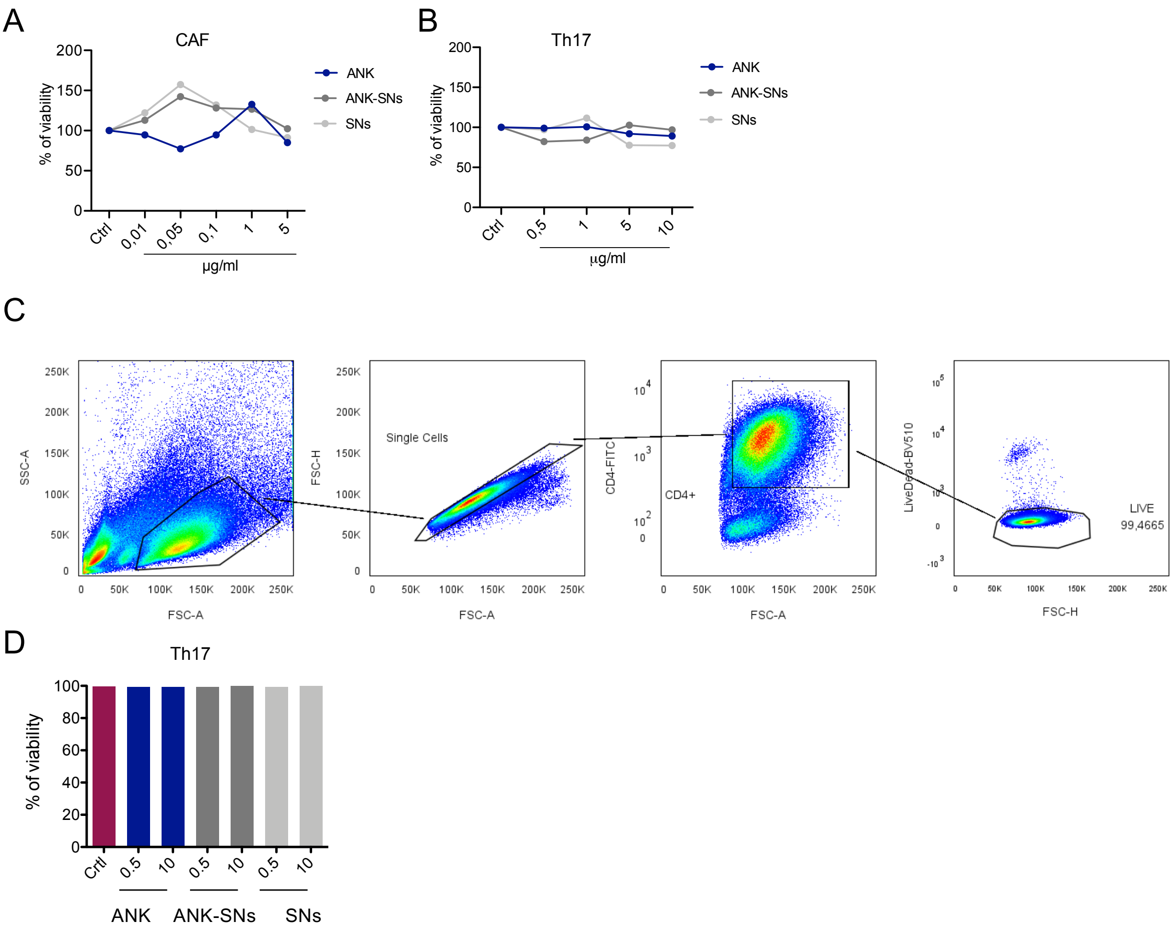

Supplementary Figure S4. **Nanosystems do not impact on cell viability**. (**A)** Percentage CAF viability detected by CellTiter colorimetric assay. CAF were activated with IL-1α plus IL-1β (Crtl), and in the presence of the indicated concentrations of ANK, ANK-SNs and SNs. (**B)** Percentage Th17 viability detected by CellTiter colorimetric assay. Th17 cells were differentiated, as described in the Materials and Methods, in the absence (Ctrl) or in the presence of the indicated concentrations of ANK, ANK-SNs and SNs. (**C-D**) Percentage of live $CD4^{+}$ T cells within Th17 cells detected by flow cytometry. (**C**) Representative plots with gating strategy. (**D**) Percentage of live $CD4^{+}$ T cells in the different experimental conditions.

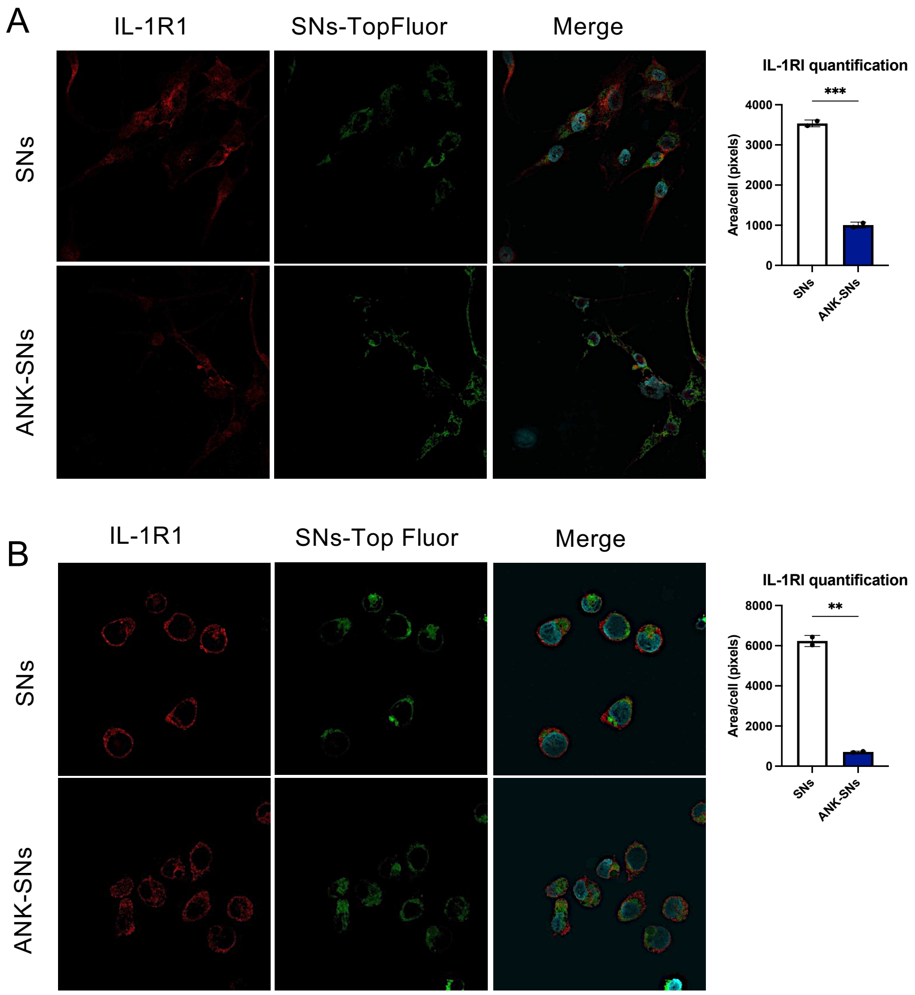

Supplementary Figure S5. ANK-SNs induce IL-1R1 internalization in both CAFs and Th17 cells. Confocal laser miscroscopy images and quantification of IL-1R1 (red) expression at the cell surface of CAFs (**A**) and Th17 (**B**) after treatment with SNs (upper panels) compared to ANK-SNs (lower panels). IL-1R1 (red), SNs (TopFluor®, green). Cell nuclei were couterstained with Hoechst (blue). Right, histograms represent the quantification of the area covered by IL-1R1 analyzed by the ImageJ software. Significance was calculated by Student's-t-test and values were considered significantly different for **p<0.01 and ***p<0.001
